# Association of Baseline Serum DKK-1 Levels with Subsequent Bone Metastasis in Stage III Breast Cancer

**DOI:** 10.3390/medicina62101922

**Published:** 2026-10-06

**Authors:** Gürkan Gül, Damla Günenç, Özlem Kutlu, Mustafa Ulaş, Aziz Aksoy, Erdinç Esenkaya, Asude Aksoy

**Affiliations:** 1 Department of Medical Oncology, Elazig Fethi Sekin City Hospital, 23280 Elazig, Türkiye; asudeaksoy@hotmail.com; 2 Department of Medical Oncology, Izmir City Hospital, 35540 Izmir, Türkiyeozlemacikerkutlu@gmail.com (Ö.K.); erdincesenkaya@hotmail.com (E.E.); 3 Department of Physiology, Faculty of Medicine, Samsun University, 55080 Samsun, Türkiye; mustafa.ulas@samsun.edu.tr; 4 Department of Bioengineering, Malatya Turgut Ozal University, 44210 Malatya, Türkiye; aksoy_aziz@hotmail.com

**Keywords:** breast cancer, bone metastasis, DKK-1, irisin, serum biomarker, ELISA, prognosis

## Abstract

*Background and Objectives*: Bone metastasis is the most frequent site of distant spread in breast cancer, yet reliable biomarkers capable of identifying patients at increased risk before metastatic disease becomes clinically evident remain limited. We investigated whether baseline serum Dickkopf-1 (DKK-1) and irisin levels were associated with subsequent bone and liver metastasis in patients with stage III breast cancer. *Materials and Methods*: Sixty-three patients with stage III breast cancer were retrospectively included. DKK-1 and irisin concentrations were measured using enzyme-linked immunosorbent assay in archived serum samples collected at the time of initial diagnosis, before the start of definitive treatment. Associations between biomarker levels and subsequent metastatic outcomes were evaluated using maximally selected rank statistics for cut-off determination, Kaplan–Meier survival analysis, restricted cubic spline analysis, and Cox proportional hazards regression models. *Results*: During follow-up, 29 patients developed bone metastasis. Baseline serum DKK-1 levels were significantly higher in patients who subsequently developed bone metastasis than in those who remained metastasis-free (*p* = 0.005), whereas irisin levels showed no significant difference. An exploratory optimal DKK-1 cut-off value of 66.75 pg/mL was identified. High baseline DKK-1 remained independently associated with bone metastasis-free survival after multivariable Cox proportional hazards regression including T stage, histological grade, Ki-67 index, lymphovascular invasion, irisin, ALP and CA15-3 (HR = 7.48, 95% CI 1.67–33.43, *p* = 0.008). Elevated baseline ALP was also independently associated with bone metastasis-free survival (BMFS) (HR = 4.12, 95% CI 1.35–12.53, *p* = 0.013). Neither DKK-1 nor irisin was significantly associated with subsequent liver metastasis. *Conclusions*: Elevated baseline serum DKK-1, but not irisin, was independently associated with shorter BMFS in patients with stage III breast cancer. Baseline DKK-1 may represent a candidate biomarker for identifying patients at increased risk of skeletal metastasis; however, these exploratory findings require external validation in prospective multicenter studies before any clinical implementation can be considered.

## 1. Introduction

Breast cancer remains the most frequently diagnosed malignancy among women worldwide and continues to be a leading cause of cancer-related mortality. Despite substantial advances in screening programs, molecular characterization, and systemic treatment strategies, the majority of breast cancer-related deaths are still attributable to distant metastatic dissemination rather than the primary tumor itself. Consequently, identifying the biological mechanisms underlying metastatic progression and developing reliable biomarkers capable of identifying high-risk patients at an early stage have become major priorities in contemporary breast cancer research [1,2].

Bone is the most common site of distant metastasis in breast cancer and is associated with skeletal-related events, including pain, pathological fractures, spinal cord compression, and hypercalcemia, all of which substantially impair quality of life and survival. Bone metastasis results from complex interactions between disseminated tumor cells and the bone microenvironment. Among the molecular pathways involved, Wnt/β-catenin signaling has emerged as a key regulator of bone remodeling and metastatic progression [1,2].

The Wnt/β-catenin signaling pathway plays a central role in both bone homeostasis and cancer progression. Dickkopf-1 (DKK-1), a potent extracellular antagonist of canonical Wnt signaling, suppresses osteoblast differentiation, reduces bone formation, and promotes osteoclast-mediated bone resorption [3]. Beyond its role in bone remodeling, DKK-1 has been implicated in epithelial–mesenchymal transition, tumor invasion, metastatic colonization, therapeutic resistance, and cancer stem cell maintenance, supporting its involvement in metastatic progression [4,5].

Increasing evidence supports the involvement of DKK-1 in breast cancer metastasis. Elevated circulating DKK-1 levels have been reported in patients with established bone metastases, and experimental studies have shown that DKK-1 promotes osteolytic niche formation by suppressing osteoblast activity and remodeling the bone microenvironment [3,6]. DKK-1 has also been implicated in organ-specific metastatic dissemination, immune evasion through suppression of natural killer cell activity, and tumor progression via CKAP4/PI3K-AKT signaling, highlighting its multifaceted role in breast cancer progression [5,7,8].

Clinical studies have shown that elevated circulating DKK-1 levels are associated with advanced disease, lymph node involvement, aggressive clinicopathological features, and poorer survival [9,10]. In addition, increased DKK-1 expression has been linked to biologically aggressive breast cancer subtypes and may predict response to neoadjuvant therapy [11]. These findings have stimulated growing interest in identifying additional circulating biomarkers that may complement DKK-1 in predicting metastatic progression and disease outcomes.

Irisin, a myokine derived from fibronectin type III domain-containing protein 5 (FNDC5), has also emerged as a potential biomarker in cancer. Experimental studies suggest that irisin suppresses tumor proliferation, migration, invasion, and epithelial–mesenchymal transition through modulation of PI3K/Akt, STAT3, and mTOR signaling [12,13]. Clinical studies have reported inconsistent associations between circulating irisin levels and breast cancer characteristics, highlighting the need for further investigation [14]. However, its role in metastatic progression, particularly in predicting organ-specific metastatic spread, remains poorly defined. Current clinical evidence is heterogeneous, and further investigations are required to clarify the prognostic and predictive significance of irisin in breast cancer [12,13]. Beyond its anti-tumour properties, irisin has also been proposed to exert protective effects within the bone microenvironment, with potential roles in modulating pathways implicated in bone resorption and skeletal metastatic colonization [12,13]. Furthermore, circulating irisin levels have been reported to be lower in breast cancer patients compared with healthy controls, suggesting systemic dysregulation of the muscle–bone axis [14].

However, most previous studies have evaluated DKK-1 in patients with established metastatic disease, while relatively little is known about the relationship between baseline DKK-1 and irisin levels and the later development of metastasis in patients who are initially free of metastatic disease. Therefore, this study aimed to examine whether DKK-1 and irisin levels measured in archived serum samples collected at the time of initial diagnosis were associated with the subsequent development of bone and liver metastases during follow-up in patients with stage III breast cancer.

## 2. Materials and Methods

### 2.1. Study Population

This retrospective study screened 127 consecutive female patients with newly diagnosed stage III breast cancer who were treated at the institutional oncology clinic between February 2007 and December 2022. Patients with distant metastatic disease at diagnosis or a previous history of malignancy were excluded. For biomarker analyses, archived pretreatment serum samples that had been collected at the time of initial diagnosis and stored under appropriate conditions were retrospectively identified. Patients without an available pretreatment archived serum sample or those with incomplete follow-up data were excluded. After applying the eligibility criteria, 63 patients were included in the final analysis.

Clinical, pathological, laboratory, treatment, and follow-up data were retrieved from electronic medical records. All patients received guideline-concordant multimodal treatment according to contemporary institutional protocols and international guideline recommendations applicable at the time of diagnosis. Anthracycline- and taxane-based chemotherapy was administered in either the neoadjuvant (65%) or adjuvant (35%) setting according to clinical presentation and multidisciplinary team decision. Patients with HER2-positive disease received trastuzumab-based anti-HER2 therapy for a total duration of one year, irrespective of whether treatment was initiated in the neoadjuvant or adjuvant setting. HER2-positive disease included both hormone receptor-positive/HER2-positive and hormone receptor-negative/HER2-positive tumors. No patient received pertuzumab. Hormone receptor-positive premenopausal patients received ovarian function suppression combined with an aromatase inhibitor, whereas postmenopausal patients received aromatase inhibitor-based endocrine therapy. Accordingly, endocrine therapy was administered to all hormone receptor-positive patients irrespective of HER2 status. Planned chemotherapy was completed by 61 of 63 patients (96.8%), whereas two patients discontinued treatment because of treatment-related toxicity. All patients underwent definitive breast surgery followed by adjuvant radiotherapy according to contemporary treatment guidelines.

The study was conducted in accordance with the Declaration of Helsinki and was initially approved by the Non-Interventional Clinical Research Ethics Committee of Fırat University (decision no. 2021/01-21, 14 January 2021). An additional approval covering the retrospective evaluation of DKK-1 and metastatic outcomes in the entire study cohort (2007–2022) was obtained from the Non-Interventional Clinical Research Ethics Committee of Elazığ Fethi Sekin City Hospital (decision no. 2026/30-25, 21 May 2026). Written informed consent was obtained from all participants for the research use of their previously collected and archived biological samples.

### 2.2. Blood Sample Collection and Biomarker Measurement

Archived pretreatment serum samples were used for biomarker analyses. The samples had originally been obtained within 7 days after breast cancer diagnosis and before surgical intervention and/or initiation of neoadjuvant systemic therapy. At the time of collection, blood samples had been processed according to the institutional sample-processing protocol in place at that time. As part of this protocol, aprotinin (20–30 μL per mL of blood) was added immediately after blood collection to minimize proteolytic degradation. Following centrifugation, serum samples were aliquoted into cryovials and stored at −80 °C until biomarker analysis. Each aliquot underwent a single freeze–thaw cycle prior to biomarker measurement.

Serum concentrations of Dickkopf-1 (DKK-1) and irisin were measured using commercially available enzyme-linked immunosorbent assay (ELISA) kits according to the manufacturers’ instructions. DKK-1 levels were determined using the Human DKK-1/Dickkopf-1 ELISA Kit PicoKine^®^ (Boster Biological Technology, Pleasanton, CA, USA; Catalog No. EK0867), whereas irisin concentrations were measured using the Human Irisin/FNDC5 ELISA Kit PicoKine^®^ (Boster Biological Technology, Pleasanton, CA, USA; Catalog No. EK2385-1). To minimize inter-assay variability, all serum samples were analyzed in the same analytical batch. All samples were assayed in duplicate, and the mean value of the two measurements was used for statistical analyses. Laboratory personnel performing the ELISA assays were blinded to all clinicopathological characteristics and patient outcomes.

For ELISA procedures, washing steps were performed using an automated microplate washer (ELx50, BioTek Instruments, Winooski, VT, USA), and absorbance values were measured using a microplate reader (ChroMate P4300, Awareness Technology Instruments, Palm City, FL, USA). Analytical performance characteristics, including assay sensitivity, measurement range, and precision, were in accordance with the manufacturers’ specifications.

### 2.3. Outcome Definition

Bone metastasis-free survival (BMFS) was defined as the time from breast cancer diagnosis to the first confirmed bone metastasis or the date of last follow-up. When bone metastasis was clinically suspected during follow-up, positron emission tomography/computed tomography (PET/CT), magnetic resonance imaging (MRI), computed tomography (CT), or bone scintigraphy was performed as clinically indicated. In patients with suspicious findings or findings consistent with bone metastasis on MRI, CT, or bone scintigraphy, PET/CT was used for further evaluation and confirmation; in some patients, bone metastasis was detected directly by PET/CT. All 29 patients who developed bone metastasis had PET/CT findings consistent with bone metastasis. PET/CT images were reviewed by two experienced nuclear medicine physicians, who confirmed the presence of bone metastasis. In addition, bone metastasis was histopathologically confirmed in 3 patients (10.3%). Follow-up imaging was not performed at prespecified intervals but was obtained as part of routine clinical practice based on symptoms, clinical findings, or suspicion of disease recurrence.

### 2.4. Statistical Analysis

Statistical analyses were performed using IBM SPSS Statistics software (Version 26.0; IBM Corp., Armonk, NY, USA). Maximally selected rank statistics, restricted cubic spline analysis, and time-dependent ROC analyses were performed using R software (R Foundation for Statistical Computing, Vienna, Austria; https://www.R-project.org/, accessed on 5 October 2026) with the maxstat, survival, rms, and timeROC packages. Continuous variables were expressed as mean ± standard deviation (SD) or median (interquartile range [IQR]) according to data distribution, whereas categorical variables were presented as frequencies and percentages.

The normality of continuous variables was assessed using visual methods (histograms and Q-Q plots) and the Kolmogorov–Smirnov test. Comparisons between patients who developed bone metastasis and those who did not were performed using the independent-samples *t*-test or Mann–Whitney U test, as appropriate. Categorical variables were compared using the chi-square test or Fisher’s exact test.

Correlations between continuous variables were evaluated using Spearman’s rank correlation analysis.

Maximally selected rank statistics were applied to determine optimal cut-off values for DKK-1, irisin, CA15-3, alkaline phosphatase (ALP), calcium, and albumin. The Ki-67 index was dichotomized according to the St. Gallen 2015 international consensus [15] as low (≤20%) versus high (>20%). Categorical clinical variables were grouped as follows: age (≤40 vs. >40 years), molecular subtype (HR+/HER2− vs. HER2+ [HR+/−] or triple-negative), T stage (T1–T2 vs. T3–T4), N stage (N1 vs. N2–N3), histological grade (grade 1–2 vs. grade 3), and lymphovascular invasion (negative vs. positive). Patients were subsequently categorized into low and high groups according to the derived cut-off values for all survival analyses.

Survival curves were estimated using the Kaplan–Meier method and compared using the log-rank test. Univariable Cox proportional hazards regression analyses were performed to identify factors associated with bone metastasis-free survival (BMFS). Variables demonstrating statistical significance in univariable analyses (*p* < 0.05) were entered into a multivariable Cox proportional hazards regression model. Hazard ratios (HRs) with corresponding 95% confidence intervals (95% CIs) were reported, and the model’s discriminatory performance was assessed by Harrell’s concordance index (C-index).

To evaluate the association between baseline DKK-1 as a continuous biomarker and bone metastasis-free survival (BMFS), univariable Cox proportional hazards regression analysis was also performed. Median follow-up duration was estimated using the reverse Kaplan–Meier method. A two-sided *p* value < 0.05 was considered statistically significant. Additionally, to characterize the functional form of the DKK-1–BMFS relationship, a restricted cubic spline (RCS) Cox proportional hazards model with three degrees of freedom was applied to continuous DKK-1 values, and the resulting dose–response curve was assessed graphically. The time-dependent discriminatory performance of baseline DKK-1 for bone metastasis, both as a continuous variable and dichotomized at the 66.75 pg/mL cut-off, was evaluated using cumulative/dynamic time-dependent receiver operating characteristic (ROC) curves with inverse probability of censoring weighting. The area under the curve (AUC) was calculated at 1, 3, and 5 years, and 95% CIs were obtained by bootstrap resampling (2000 replicates).

## 3. Results

A total of 63 patients with non-metastatic stage III breast cancer were included in the study. During follow-up, 29 patients developed bone metastasis, whereas 34 patients remained free of bone metastasis. The median follow-up duration for the entire cohort was 123 months (95% CI, 116.3–129.7 months).

No significant differences were observed between patients who developed bone metastasis and those who remained free of bone metastasis with respect to age, menopausal status, histological subtype, grade, molecular subtype, N stage, systemic treatment or lumbar spine DEXA classification (all *p* > 0.05). T stage differed significantly between groups, with T3–T4 tumours more prevalent among patients who developed bone metastasis (*p* = 0.003). Ki-67 index categorised as high (>20%) was also more frequent in the bone metastasis group (*p* = 0.048). Lymphovascular invasion (LVI) positivity was higher in patients who subsequently developed bone metastasis (*p* < 0.001). Among laboratory parameters, baseline serum CA15-3 levels were significantly higher in patients who subsequently developed bone metastasis than in those who remained metastasis-free (27.47 ± 23.99 vs. 16.83 ± 11.07 U/mL, *p* = 0.039). Likewise, baseline serum DKK-1 levels were significantly higher in patients who developed bone metastasis during follow-up than in those who did not (124.52 ± 94.13 vs. 108.27 ± 106.10 pg/mL, *p* = 0.005). No significant differences were observed for serum calcium, albumin, alkaline phosphatase (ALP), or irisin levels (Table 1).

Correlation analysis demonstrated a significant positive association between serum DKK-1 and CA15-3 levels (*r* = 0.357, *p* = 0.004; Supplementary Table S1), indicating that higher circulating DKK-1 concentrations were associated with increased tumor marker levels.

**Figure 1:**
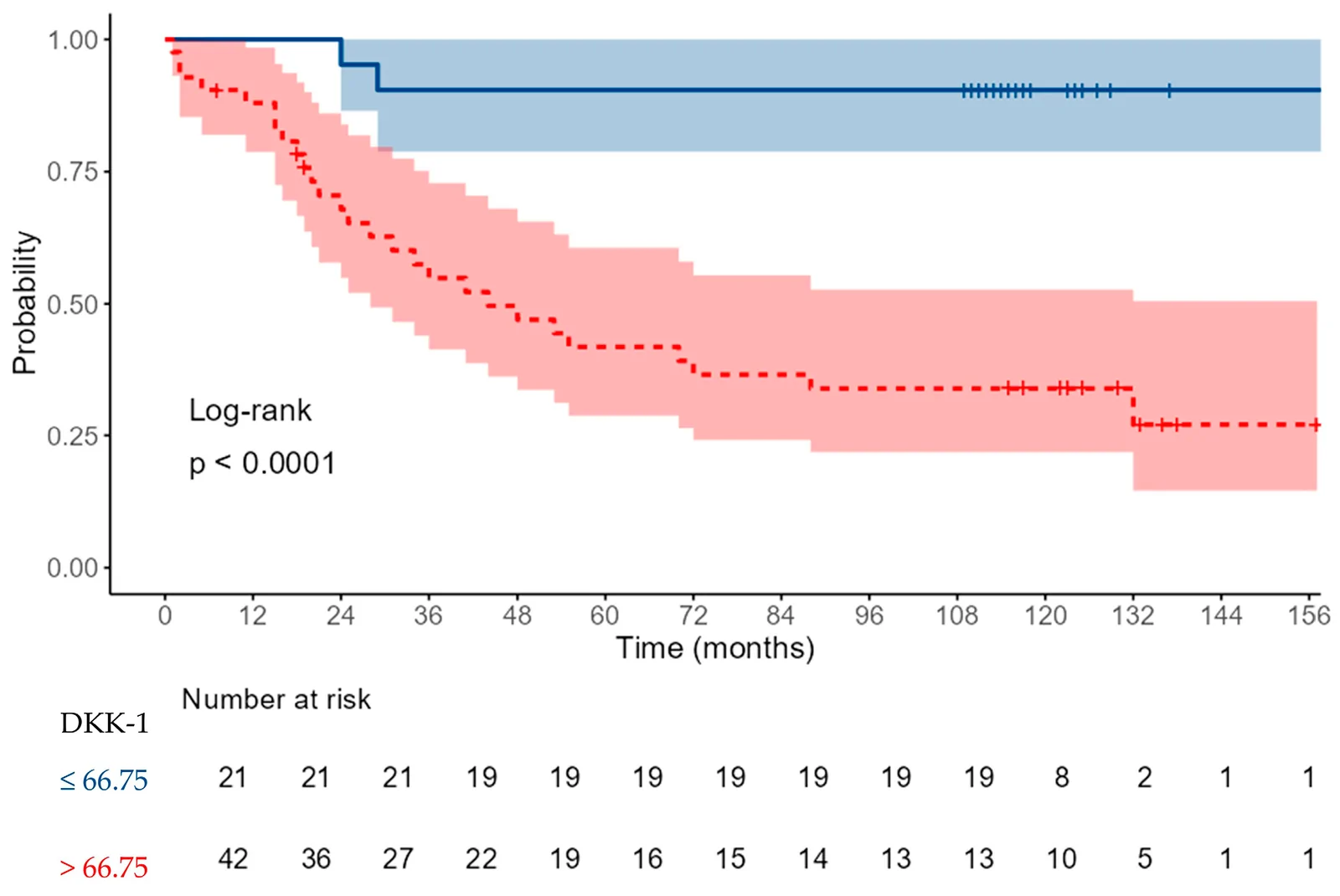
Kaplan–Meier curves for bone metastasis-free survival according to baseline serum DKK-1 (≤66.75 vs. >66.75 pg/mL). The solid blue line represents patients with low DKK-1 (≤66.75 pg/mL), and the dashed red line represents patients with high DKK-1 (>66.75 pg/mL). Shaded areas indicate 95% confidence intervals, and tick marks indicate censored observations. The number of patients at risk is shown below the plot. The *p* value was calculated using the log-rank test.

Maximally selected rank statistics identified optimal cut-off values for continuous variables: DKK-1, 66.75 pg/mL; irisin, 76.7 ng/mL; CA15-3, 25.0 U/mL; ALP, 87.0 U/L; calcium, 9.70 mg/dL; and albumin, 4.40 g/dL (Supplementary Table S2 and Figures S1–S5). Patients were subsequently categorized into low and high groups according to these cut-off values for survival and regression analyses. Kaplan–Meier analysis demonstrated significantly shorter bone metastasis-free survival (BMFS) among patients with high baseline DKK-1 levels than among those with low levels (log-rank *p* < 0.001) (Figure 1). The median BMFS was not reached in the low DKK-1 group (1-year BMFS: 100.0%; 3-year BMFS: 90.5%; 5-year BMFS: 90.5%), whereas it was 44 months (95% CI, 21–67 months) in the high DKK-1 group (1-year BMFS: 88.0%; 3-year BMFS: 54.8%; 5-year BMFS: 41.8%).

In univariable Cox proportional hazards regression, baseline serum DKK-1 levels > 66.75 pg/mL were strongly associated with shorter BMFS (HR = 10.60, 95% CI 2.51–44.75, *p* = 0.001). Other variables associated with BMFS included ALP (HR = 4.88, 95% CI 1.97–12.07, *p* = 0.001), lymphovascular invasion (HR = 3.89, 95% CI 1.72–8.83, *p* = 0.001), irisin (HR = 3.01, 95% CI 1.44–6.29, *p* = 0.003), T stage (HR = 3.57, 95% CI 1.36–9.39, *p* = 0.010), CA15-3 (HR = 2.83, 95% CI 1.34–5.98, *p* = 0.007), Ki-67 index (HR = 2.42, 95% CI 1.10–5.33, *p* = 0.028), and histological grade (HR = 2.17, 95% CI 1.01–4.63, *p* = 0.046). Age group, menopausal status, histological type, molecular subtype, N stage, lumbar spine DEXA classification, serum calcium, and albumin were not significantly associated with BMFS. Multivariable Cox proportional hazards regression, adjusting simultaneously for DKK-1, ALP, CA15-3, T stage, histological grade, Ki-67 index, lymphovascular invasion, and irisin, confirmed that high baseline serum DKK-1 remained independently associated with shorter BMFS (HR = 7.48, 95% CI 1.67–33.43, *p* = 0.008). Elevated ALP was also independently associated with BMFS (HR = 4.12, 95% CI 1.35–12.53, *p* = 0.013). The overall discriminatory performance of the multivariable model was good (C-index = 0.822, SE = 0.033; likelihood ratio test *p* < 0.001) (Table 2). The proportional hazards assumption was not violated for the DKK-1 group (Schoenfeld residual test, *p* = 0.752; Supplementary Figure S6).

When DKK-1 was analyzed as a continuous variable, no significant association with BMFS was observed in the univariable Cox regression (HR = 1.001, 95% CI 0.997–1.004, *p* = 0.655).

**Figure 2:**
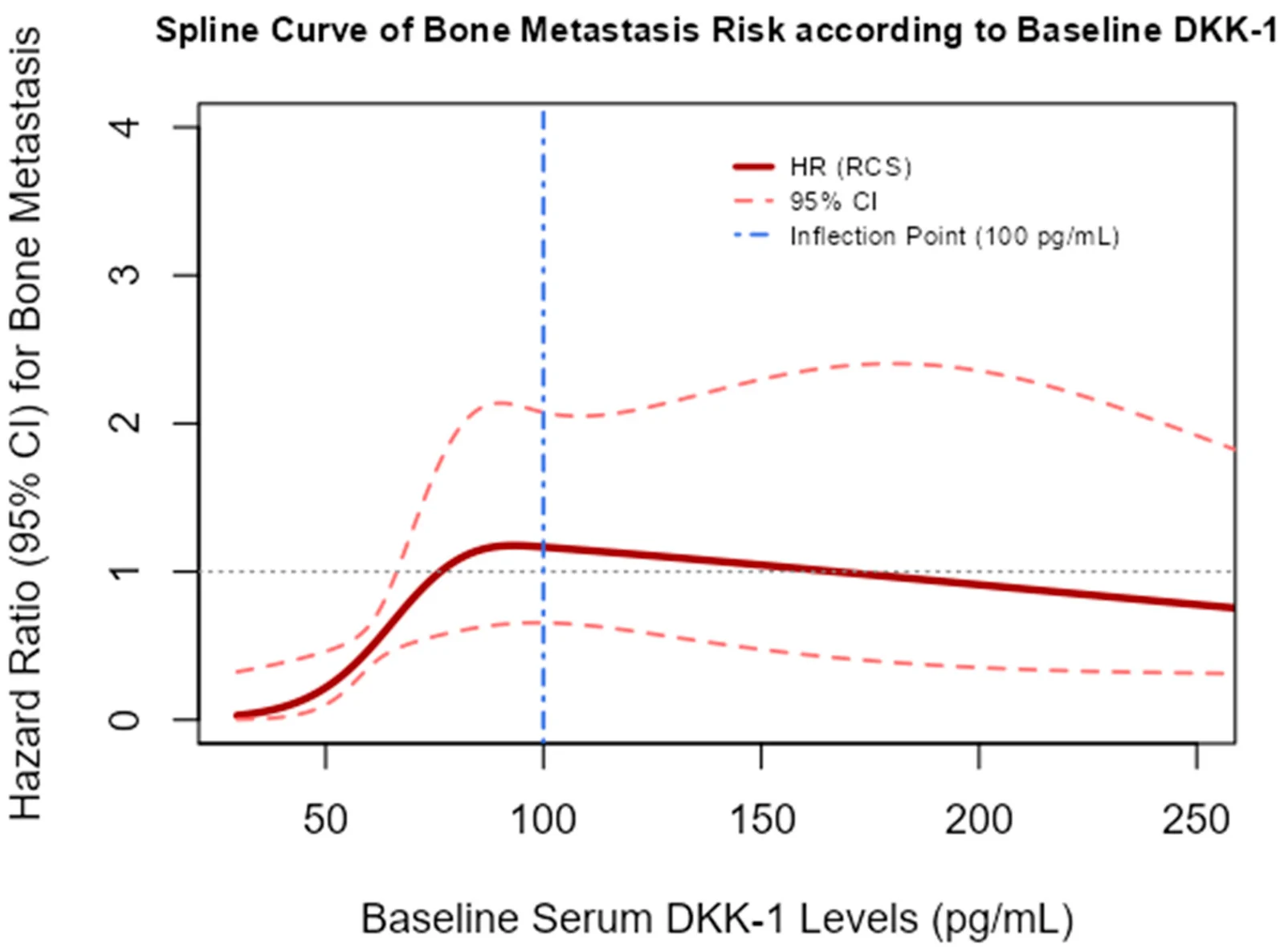
Restricted cubic spline curve showing the non-linear relationship between baseline serum DKK-1 levels and bone metastasis hazard ratio (HR). The solid line indicates the estimated HR, with dashed lines representing 95% confidence intervals. The vertical blue line marks the inflection point at approximately 100 pg/mL, and the horizontal line indicates the reference HR of 1.0.

To further characterize the functional form of the DKK-1–BMFS relationship, a restricted cubic spline (RCS) Cox proportional hazards model with three degrees of freedom was applied to the continuous DKK-1 variable. The overall spline model demonstrated a statistically significant association between continuous DKK-1 concentration and BMFS (likelihood ratio test: χ^2^ = 12.5, df = 3, *p* = 0.006), indicating that the DKK-1–BMFS relationship departed significantly from linearity. Visual inspection of the dose–response curve revealed a non-linear, threshold-type pattern: the estimated hazard ratio rose steeply at DKK-1 concentrations approaching and exceeding 66.75 pg/mL, reached a peak at approximately 100 pg/mL, and subsequently reached a plateau at higher concentrations (Figure 2). In time-dependent ROC analysis, the AUC of continuous DKK-1 was 0.649 (95% CI 0.454–0.830), 0.573 (95% CI 0.429–0.720), and 0.665 (95% CI 0.524–0.804) at 1, 3, and 5 years, respectively, whereas the corresponding AUCs of dichotomized DKK-1 were 0.684 (95% CI 0.624–0.746), 0.686 (95% CI 0.586–0.787), and 0.731 (95% CI 0.623–0.830) (Supplementary Figure S7). The modest time-dependent discrimination of continuous DKK-1 is consistent with the non-linear, threshold-type relationship observed in the spline analysis; however, the AUCs of the dichotomized variable may be optimistic because the cut-off was derived in the same cohort, and the 1-year estimates were based on only five events.

During follow-up, 14 patients (22.2%) developed liver metastasis. Baseline albumin levels were significantly higher in these patients (*p* = 0.021), whereas no significant differences were observed in other clinicopathological or laboratory parameters, including DKK-1 and irisin (all *p* > 0.05) (Table 3).

## 4. Discussion

In our study, DKK-1 was evaluated as a prognostic and risk-stratification biomarker, reflecting its potential to identify patients at higher risk of skeletal metastasis at the time of initial diagnosis, although this potential remains to be confirmed in independent cohorts. Baseline serum DKK-1 levels were higher in patients who subsequently developed bone metastases than in those who remained free of skeletal disease during follow-up. Maximally selected rank statistics identified an optimal DKK-1 cut-off of 66.75 pg/mL, and patients with DKK-1 levels above this threshold had significantly shorter BMFS. Serum DKK-1 levels also showed a positive correlation with CA15-3 and remained independently associated with BMFS in a multivariable Cox regression model. Overall, our results support a possible relationship between higher circulating DKK-1 levels and an increased tendency for subsequent skeletal metastasis.

An important analytical observation is the discordance between the linear continuous and non-linear spline-based modeling of DKK-1. When DKK-1 was modeled as a linear predictor in univariable Cox proportional hazards regression, no statistically significant association with BMFS was detected. In contrast, restricted cubic spline analysis demonstrated a statistically significant overall DKK-1–BMFS relationship with a non-linear dose–response pattern. The spline-derived dose–response curve revealed a threshold-type effect. The threshold phenomenon is consistent with the biological role of DKK-1 as an inhibitor of canonical Wnt/β-catenin signaling. Exceeding a functional concentration threshold may be necessary to sufficiently suppress osteoblast activity and prime the bone microenvironment for metastatic colonization. However, the biological effects of DKK-1 appear to be context-dependent; in experimental models, DKK-1 promoted bone metastasis while suppressing lung metastasis of breast cancer cells [7], and stroma-derived DKK-1 has been shown to modulate antitumor immunity [8]. As circulating DKK-1 may also originate from non-tumor sources, our observational findings indicate an association rather than a causal relationship.

Our findings are consistent with previous clinical studies showing that elevated DKK-1 expression is associated with aggressive clinicopathological features, advanced disease, metastatic spread, and poorer survival in breast cancer [16,17,18]. In addition, increased DKK-1 expression has been reported even in early-stage disease, suggesting that DKK-1 may reflect intrinsic tumor biology rather than simply tumor burden [17]. Unlike most previous studies, which evaluated patients with established metastases, we measured DKK-1 using archived serum samples collected at the time of initial diagnosis, before metastatic disease became clinically evident. Our findings therefore suggest that higher baseline DKK-1 levels may be present before the clinical detection of bone metastasis, rather than simply reflecting established skeletal disease. If validated in larger prospective studies, baseline DKK-1 assessment could help identify patients at increased risk of skeletal metastasis before clinical or radiological detection; at present, however, DKK-1 measurement should not be used to guide surveillance or treatment decisions.

We also observed a positive correlation between serum DKK-1 and CA15-3 levels. Although modest, this finding suggests that elevated DKK-1 may be associated with increased tumor activity and metastatic potential. Previous studies have linked elevated CA15-3 levels to disease recurrence and distant metastasis in breast cancer [19,20]. Although serum DKK-1 showed a positive correlation with CA15-3, only DKK-1 remained independently associated with BMFS after multivariable adjustment. The multivariable Cox proportional hazards model incorporated DKK-1 together with ALP, CA15-3, T stage, histological grade, Ki-67 index, lymphovascular invasion, and irisin, and DKK-1 retained independent prognostic significance within this comprehensive model. The incremental predictive value of DKK-1 over an even broader panel of molecular and imaging biomarkers remains to be established in larger prospective studies.

The role of irisin in cancer remains controversial. Although experimental studies suggest that irisin may inhibit tumor growth and metastatic spread, clinical findings have been inconsistent [21,22,23]. Reported associations between circulating irisin levels and prognosis vary across studies, likely reflecting differences in patient populations, tumor subtypes, and assay methodologies. In line with this heterogeneous evidence, although baseline serum irisin levels were associated with shorter BMFS in univariable Cox proportional hazards analysis (HR = 3.01, 95% CI 1.44–6.29, *p* = 0.003), this association was no longer statistically significant after multivariable adjustment (HR = 2.08, 95% CI 0.80–5.45, *p* = 0.135), suggesting that the univariable association was largely explained by other prognostic factors included in the multivariable model.

With respect to liver metastasis, baseline serum DKK-1 and irisin levels did not differ significantly between patients who developed liver metastasis and those who did not. Given the limited number of liver metastasis events in this cohort, formal survival analysis was not performed. Accordingly, these findings should be interpreted with caution and are considered hypothesis-generating.

Several strengths of this study should be considered. First, DKK-1 and irisin levels were measured using archived serum samples collected at the time of initial diagnosis and before the start of definitive treatment. As these samples were obtained before the development of metastatic disease, they allowed us to examine whether baseline biomarker levels were associated with subsequent metastasis rather than with already established metastatic disease. This approach also reduced the possibility that the biomarker levels were influenced by metastatic progression itself. Second, DKK-1 was evaluated both in relation to the development of bone metastasis and to bone metastasis-free survival, providing complementary information on its association with skeletal progression. In addition, evaluating bone and liver metastases separately allowed us to explore whether the observed associations differed according to the site of metastatic spread. The association between DKK-1, subsequent bone metastasis, and CA15-3 further supports the internal consistency of our findings. Finally, the use of serum biomarkers may have practical relevance because they can be assessed using readily accessible biological samples. Further prospective studies in larger, independent cohorts are needed to determine whether baseline DKK-1 could contribute to identifying patients at increased risk of subsequent bone metastasis.

This study has several limitations. Its retrospective single-center design may have introduced selection bias and residual confounding. The use of archived serum samples collected over an extended period may also have introduced some preanalytical variability, despite standardized processing and storage at −80 °C. In addition, the relatively small sample size and limited number of events reduced the statistical power, particularly for subgroup and multivariable analyses. In particular, the small number of events in the low DKK-1 group resulted in a wide confidence interval for the adjusted HR, and the magnitude of this estimate should therefore be interpreted with caution. The low number of triple-negative breast cancer cases also prevented meaningful subtype-specific analyses. The underrepresentation of triple-negative breast cancer cases is a notable limitation, as TNBC may differ from hormone receptor-positive and HER2-positive subtypes in DKK-1-mediated osteolytic mechanisms and patterns of metastatic tropism. Biomarker measurements were performed only once using pretreatment serum samples; therefore, changes in DKK-1 and irisin levels during treatment and follow-up could not be evaluated. Furthermore, the study spanned more than 15 years, during which breast cancer treatment strategies changed considerably; treatment-era differences may therefore have influenced metastatic outcomes.

Although histopathological confirmation remains the gold standard for the definitive diagnosis of bone metastasis, biopsy of every suspected bone lesion is not routinely feasible or clinically indicated in practice. All patients who developed bone metastasis had PET/CT findings consistent with skeletal metastatic disease, whereas histopathological confirmation was available in only three patients. Furthermore, because of the retrospective design, follow-up imaging was not performed at prespecified intervals but was obtained based on symptoms, clinical findings, or suspicion of disease recurrence. This may have introduced detection bias, particularly with respect to the timing of bone metastasis detection. Asymptomatic bone metastases may therefore have been detected later in the disease course or remained undetected in patients who did not undergo imaging, potentially leading to underestimation of skeletal progression.

Finally, because the cohort mainly consisted of hormone receptor-positive and HER2-positive tumors, the findings may not be fully generalizable to other breast cancer populations. The optimal DKK-1 cut-off was derived from the study cohort and was not externally validated; therefore, the proposed threshold should be considered exploratory. Prospective multicenter studies with larger cohorts, standardized imaging protocols, and histopathological confirmation when clinically appropriate are needed to validate these findings and better define the prognostic value of baseline DKK-1.

## 5. Conclusions

In conclusion, elevated baseline serum DKK-1 levels were independently associated with subsequent bone metastasis and shorter bone metastasis-free survival in patients with stage III breast cancer, whereas serum irisin levels showed no significant association with metastatic outcomes. An exploratory DKK-1 cut-off of 66.75 pg/mL was identified using maximally selected rank statistics; although the magnitude of the association should be interpreted with caution, these findings support a potential role for DKK-1 as a risk-stratification biomarker for identifying patients at increased risk of future skeletal metastasis. Nevertheless, because the proposed cut-off was derived from a relatively small retrospective cohort, prospective multicenter studies are required to externally validate these findings before any clinical implementation can be considered.

## Figures and Tables

**Table 1:** Baseline clinicopathological characteristics of patients according to subsequent bone metastasis development.

Variable	Bone Metastasis (*n* = 29)	No Bone Metastasis (*n* = 34)	*p* Value
Age, years, mean ± SD	48.13 ± 13.4	49.02 ± 10.84	0.772
Median	47	47	
Menopausal status, *n* (%)			0.504
Pre-menopausal	20 (69%)	26 (76.5%)	
Menopausal	9 (31%)	8 (23.5%)	
Histological type, *n* (%)			0.165
Invasive ductal carcinoma	22 (75.9%)	31 (91.2%)	
Other histologies	7 (24.1%)	3 (8.8%)	
Histological grade, *n* (%)			0.155
Grade 1–2	11 (37.9%)	19 (55.9%)	
Grade 3	18 (62.1%)	15 (44.1%)	
Molecular subtype, *n* (%)			0.620
HR+/HER2−	12 (41.4%)	12 (35.3%)	
HER2+ or Triple-negative	17 (58.6%)	22 (64.7%)	
T stage, *n* (%)			0.003
T1–T2	5 (17.2%)	18 (52.9%)	
T3–T4	24 (82.8%)	16 (47.1%)	
N stage, *n* (%)			0.741
N1	14 (48.3%)	15 (44.1%)	
N2–N3	15 (51.7%)	19 (55.9%)	
Ki-67 index (%)			0.048
Low (≤20)	9 (31%)	19 (55.9%)	
High (>20)	20 (69%)	15 (44.1%)	
Lymphovascular invasion, *n* (%)			<0.001
Negative	8 (27.6%)	25 (73.5%)	
Positive	21 (72.4%)	9 (26.5%)	
Systemic treatment, *n* (%)			0.857
Chemotherapy	29 (100%)	34 (100%)	
Endocrine therapy	20 (69%)	33 (97.1%)	
Anti-HER2 therapy	16 (55.2%)	21 (61.8%)	
Lumbar spine DEXA classification			0.252
Normal	10 (34.5%)	12 (35.3%)	
Osteopenia	10 (34.5%)	17 (50.0%)	
Osteoporosis	9 (31%)	5 (14.7%)	
Dickkopf-1	124.52 ± 94.13	108.27 ± 106.10	**0.005**
Median	99.22	66.10	
Irisin	87.64 ± 53.46	86.55 ± 105.71	0.095
Median	77.5	61.63	
Laboratory parameters			
CA15-3	27.47 ± 23.99	16.83 ± 11.07	0.039
Calcium	9.48 ± 0.56	9.27 ± 0.60	0.150
Albumin	4.45 ± 0.49	4.32 ± 0.41	0.308
ALP	74.82 ± 24.84	69.55 ± 38.83	0.055

ALP, alkaline phosphatase; DEXA, dual-energy X-ray absorptiometry; HER2, human epidermal growth factor receptor 2; HR+, hormone receptor-positive; SD, standard deviation. Age was compared using the independent-samples *t*-test. Categorical variables were compared using the chi-square test or Fisher’s exact test, as appropriate. Continuous laboratory parameters were compared using the Mann–Whitney U test. Both mean ± SD and median values are reported for continuous variables.

**Table 2:** Univariable and multivariable Cox proportional hazards regression analyses for bone metastasis-free survival.

Variable	Univariable		Multivariable	
	HR (95% CI)	*p*-Value	HR (95% CI)	*p*-Value
Age group				
≤40 years	Reference		—	—
>40 years	0.78 (0.35–1.77)	0.554		
Histological type				
Invasive Ductal Carcinoma	Reference		—	—
Non-ductal	1.97 (0.84–4.64)	0.119		
Molecular subtype				
HR+/HER2−	Reference		—	—
HER2+ or TNBC	0.82 (0.39–1.72)	0.599		
T stage				
T1–T2	Reference		Reference	
T3–T4	3.57 (1.36–9.39)	**0.010**	1.28 (0.38–4.33)	0.690
N stage				
N1	Reference			
N2–N3	1.05 (0.51–2.18)	0.892	—	—
Histological grade				
Grade 1–2	Reference		Reference	
Grade 3	2.17 (1.01–4.63)	**0.046**	2.68 (0.83–8.63)	0.099
Ki-67 index (%)				
Low (≤20)	Reference		Reference	
High (>20)	2.42 (1.10–5.33)	**0.028**	0.81 (0.26–2.51)	0.709
Lymphovascular invasion				
Negative	Reference		Reference	
Positive	3.89 (1.72–8.83)	**0.001**	1.80 (0.65–5.00)	0.261
Menopausal status				
Premenopausal	Reference		—	—
Postmenopausal	1.30 (0.59–2.86)	0.515		
Lumbar spine DEXA classification				
Normal	Reference			
Osteopenia	0.72 (0.30–1.73)	0.466	—	—
Osteoporosis	1.58 (0.64–3.90)	0.321		
DKK-1 (pg/mL)				
Low (≤66.75)	Reference		Reference	
High (>66.75)	10.60 (2.51–44.75)	**0.001**	7.48 (1.67–33.43)	**0.008**
Irisin (ng/mL)				
Low (≤76.7)	Reference		Reference	
High (>76.7)	3.01 (1.44–6.29)	**0.003**	2.08 (0.80–5.45)	0.135
ALP (U/L)				
Low (≤87.0)	Reference		Reference	
High (>87.0)	4.88 (1.97–12.07)	**0.001**	4.12 (1.35–12.53)	**0.013**
Calcium (mg/dL)				
Low (≤9.70)	Reference		—	—
High (>9.70)	2.04 (0.94–4.44)	0.072		
Albumin (g/dL)				
Low (≤4.40)	Reference		—	—
High (>4.40)	1.63 (0.78–3.39)	0.191		
CA15-3 (U/mL)				
Low (≤25.0)	Reference		Reference	
High (>25.0)	2.83 (1.34–5.98)	**0.007**	1.12 (0.45–2.77)	0.804

ALP, alkaline phosphatase; CI, confidence interval; DEXA, dual-energy X-ray absorptiometry; DKK-1, Dickkopf-1; HER2, human epidermal growth factor receptor 2; HR, hazard ratio; HR+, hormone receptor-positive; LVI, lymphovascular invasion; TNBC, triple-negative breast cancer. Bold values indicate statistically significant differences at a statistical significance level of ≤0.05.

**Table 3:** Baseline clinicopathological characteristics of patients according to subsequent liver metastasis development.

Variable	Liver Metastasis (*n* = 14)	No Liver Metastasis (*n* = 49)	*p* Value
Age, years, mean ± SD	48.85 ± 10.58	49.40 ± 12.36	0.333
Median	44.50	47	
Menopausal status, *n* (%)			0.741
Pre-menopausal	11 (78.6%)	35 (71.4%)	
Menopausal	3 (21.4%)	14 (28.6%)	
Histological type, *n* (%)			0.679
Invasive ductal carcinoma	11 (78.6%)	42 (85.7%)	
Non-ductal	3 (21.4%)	7 (14.3%)	
Molecular subtype, *n* (%)			0.835
HR+/HER2−	5 (35.7%)	19 (38.8%)	
HER2+ or TNBC	9 (64.3%)	30 (61.2%)	
T stage, *n* (%)			0.184
T1–T2	3 (21.4%)	20 (40.8%)	
T3–T4	11 (78.6%)	29 (59.2%)	
N stage, *n* (%)			0.736
N1	7 (50.0%)	22 (44.9%)	
N2–N3	7 (50.0%)	27 (55.1%)	
Histological grade, *n* (%)			0.136
Grade 1–2	4 (28.6%)	26 (53.1%)	
Grade 3	10 (71.4%)	23 (46.9%)	
Ki-67 index (%)			0.456
Low (≤20)	5 (35.7%)	23 (46.9%)	
High (>20)	9 (64.3%)	26 (53.1%)	
Lymphovascular invasion, *n* (%)			0.068
Negative	4 (28.6%)	29 (59.2%)	
Positive	10 (71.4%)	20 (40.8%)	
Systemic treatment, *n* (%)			0.984
Chemotherapy	14 (100.0%)	49 (100.0%)	
Endocrine therapy	10 (71.4%)	33 (67.3%)	
Anti-HER2 therapy	8 (57.1%)	29 (59.2%)	
Dickkopf-1	135.80 ± 122.50	110.02 ± 93.70	0.256
Median	85.71	72.99	
Irisin	95.67 ± 64.24	84.59 ± 90.58	0.381
Median	77.79	62.50	
Laboratory parameters			
CA15-3	25.79 ± 20.47	20.57 ± 18.36	0.546
Calcium	9.50 ± 0.54	9.33 ± 0.60	0.340
Albumin	4.53 ± 0.55	4.34 ± 0.41	0.021
ALP	69.92 ± 24.31	72.57 ± 35.27	0.836
AST	22.28 ± 8.14	20.87 ± 7.24	0.625
ALT	23.14 ± 14.11	25.84 ± 28.46	0.546

ALP, alkaline phosphatase; HER2, human epidermal growth factor receptor 2; HR+, hormone receptor-positive; SD, standard deviation. Age was compared using the independent-samples *t*-test. Categorical variables were compared using the chi-square test or Fisher’s exact test, as appropriate. Continuous laboratory parameters were compared using the Mann–Whitney U test. Both mean ± SD and median values are reported for continuous variables.

## Data Availability

The data that support the findings of this study are available from the corresponding author upon reasonable request.

## Supplementary Materials

The following supporting information can be downloaded at: https://www.mdpi.com/article/10.3390/medicina62101922/s1, Table S1: Spearman correlation matrix of baseline serum biomarkers and laboratory parameters; Table S2: Optimal cut-off values determined by maximally selected rank statistics and bone metastasis-free survival according to the resulting groups; Figures S1–S5: Maximally selected rank statistics plots for irisin, CA15-3, ALP, calcium, and albumin; Figure S6: Scaled Schoenfeld residual plot for the DKK-1 group; Figure S7: Time-dependent ROC curves for baseline serum DKK-1 at 1, 3, and 5 years.

## Abbreviations

The following abbreviations are used in this manuscript:

BMFS	Bone metastasis-free survival
CA15-3	Cancer antigen 15-3
DKK-1	Dickkopf-1
ELISA	Enzyme-linked immunosorbent assay
FNDC5	Fibronectin type III domain-containing protein 5
HER2	Human epidermal growth factor receptor 2
MRI	Magnetic resonance imaging
PET/CT	Positron emission tomography/computed tomography
