# Supplementary material for: Association of Baseline Serum DKK-1 Levels with Subsequent Bone Metastasis in Stage III Breast Cancer
Source: Medicina (Kaunas). 2026 Oct 6;62(10):1922. doi: 10.3390/medicina62101922 (PMC13706943; doi:10.3390/medicina62101922)
Supplement: Supplementary file 1 [file medicina-62-01922-s001.zip › medicina-4557224-supplementary.pdf]

Supplementary Materials

Association of Baseline Serum DKK-1 Levels with Subsequent Bone Metastasis in Stage III Breast Cancer

Gürkan Gül, Damla Günenç, Özlem Kutlu, Mustafa Ulaş, Aziz Aksoy, Erdinç Esenkaya, Asude Aksoy

Contents

Table S1. Spearman correlation matrix of baseline serum biomarkers and laboratory parameters.

Table S2. Optimal cut-off values determined by maximally selected rank statistics and bone metastasis-free survival according to the resulting groups.

Figures S1–S5. Maximally selected rank statistics plots for irisin, CA15-3, ALP, calcium, and albumin.

Figure S6. Scaled Schoenfeld residual plot for the DKK-1 group.

Figure S7. Time-dependent ROC curves for baseline serum DKK-1 at 1, 3, and 5 years.

Table S1. Spearman correlation matrix of baseline serum biomarkers and laboratory parameters (n = 63).

Variable	1	2	3	4	5	6	7
1. DKK-1 (pg/mL)	1						
2. Irisin (ng/mL)	0.235<br>(0.064)	1					
3. CA15-3 (U/mL)	**0.357****<br>**(0.004)**	0.056<br>(0.660)	1				
4. Ki-67 (%)	0.106<br>(0.408)	**0.253***<br>**(0.046)**	0.078<br>(0.542)	1			
5. Albumin (g/dL)	0.043<br>(0.739)	0.084<br>(0.512)	−0.056<br>(0.664)	0.155<br>(0.226)	1		
6. ALP (U/L)	0.142<br>(0.267)	−0.215<br>(0.091)	0.179<br>(0.160)	0.113<br>(0.378)	0.081<br>(0.530)	1	
7. Calcium (mg/dL)	−0.198<br>(0.120)	−0.219<br>(0.085)	−0.085<br>(0.510)	0.103<br>(0.423)	0.147<br>(0.250)	0.110<br>(0.390)	1

Values are Spearman's rank correlation coefficients (r_s); p values are given in parentheses. Statistically significant correlations are shown in bold. * $p < 0.05$; ** $p < 0.01$.

Abbreviations: ALP, alkaline phosphatase; CA15-3, cancer antigen 15-3; DKK-1, Dickkopf-1.

Table S2. Optimal cut-off values determined by maximally selected rank statistics and bone metastasis-free survival according to the resulting low and high groups (n = 63; 29 events).

Variable	Cut-off	Standardized log-rank statistic	Low group (≤ cut-off)			High group (> cut-off)		
			n (events)	Median BMFS, months	5-year BMFS, % (95% CI)	n (events)	Median BMFS, months	5-year BMFS, % (95% CI)
DKK-1 (pg/mL)	66.75	4.17	21 (2)	NR	90.5 (78.8–100.0)	42 (27)	44	41.8 (28.8–60.5)
Irisin (ng/mL)	76.73	3.22	42 (13)	NR	67.0 (53.8–83.4)	21 (16)	41	42.9 (26.2–70.2)
CA15-3 (U/mL)	25.0	2.61	47 (17)	NR	67.5 (55.2–82.4)	16 (12)	41	29.4 (13.0–66.6)
ALP (U/L)	87.0	2.68	54 (22)	NR	66.2 (54.7–80.2)	9 (7)	24	0.0 (—)
Calcium (mg/dL)	9.70	1.68	46 (19)	NR	66.2 (53.6–81.7)	17 (10)	29	38.2 (20.5–71.4)
Albumin (g/dL)	4.40	1.31	33 (13)	132	68.4 (53.9–86.7)	30 (16)	48	48.5 (33.3–70.5)

Optimal cut-off values were determined using maximally selected rank statistics with BMFS as the outcome. Median and 5-year BMFS were estimated using the Kaplan–Meier method; 95% CIs were calculated using the log-log transformation. The cut-off values were derived and evaluated in the same cohort and should be regarded as exploratory.

Abbreviations: ALP, alkaline phosphatase; BMFS, bone metastasis-free survival; CA15-3, cancer antigen 15-3; CI, confidence interval; DKK-1, Dickkopf-1; NR, not reached.

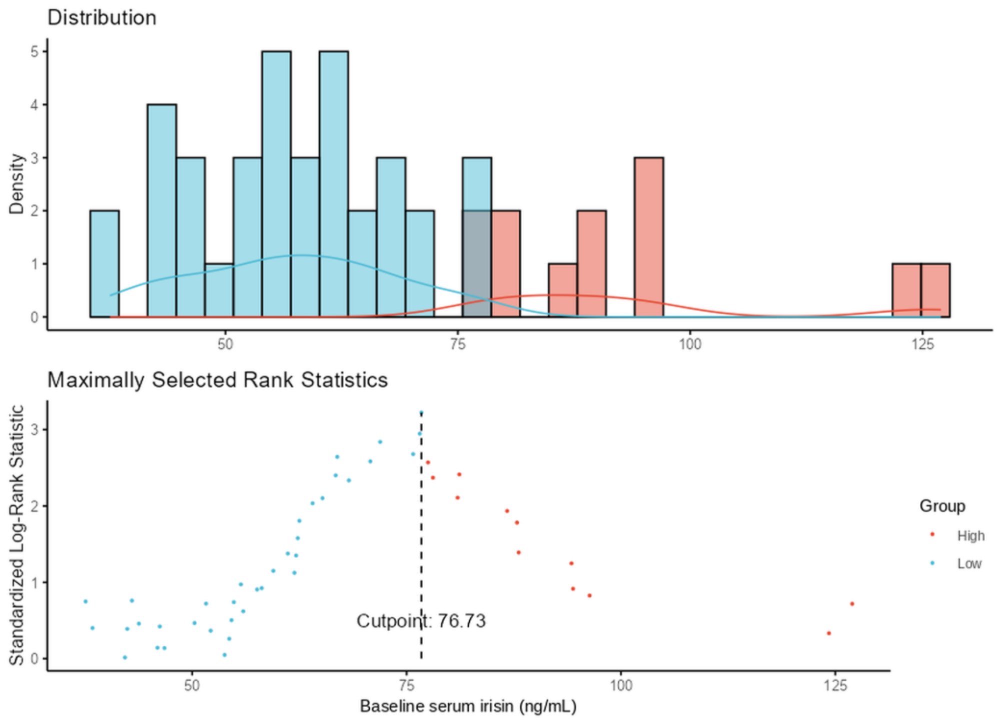

Figure S1. Determination of the optimal cut-off value for baseline serum irisin using maximally selected rank statistics. The upper panel shows the distribution of irisin values colored according to the resulting low (blue) and high (red) groups; the lower panel shows the standardized log-rank statistic for each candidate cut-off. The dashed line marks the selected cut-off (76.73 ng/mL; maximum standardized log-rank statistic = 3.22).

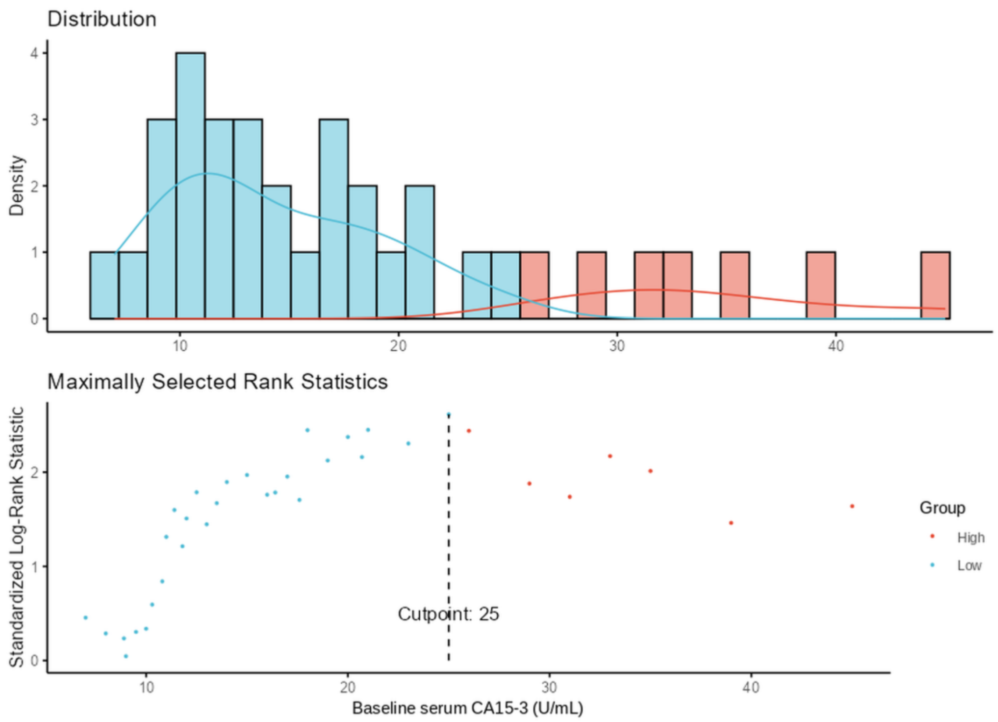

Figure S2. Determination of the optimal cut-off value for baseline serum CA15-3 using maximally selected rank statistics. Panels as in Figure S1. The dashed line marks the selected cut-off (25.0 U/mL; maximum standardized log-rank statistic = 2.61).

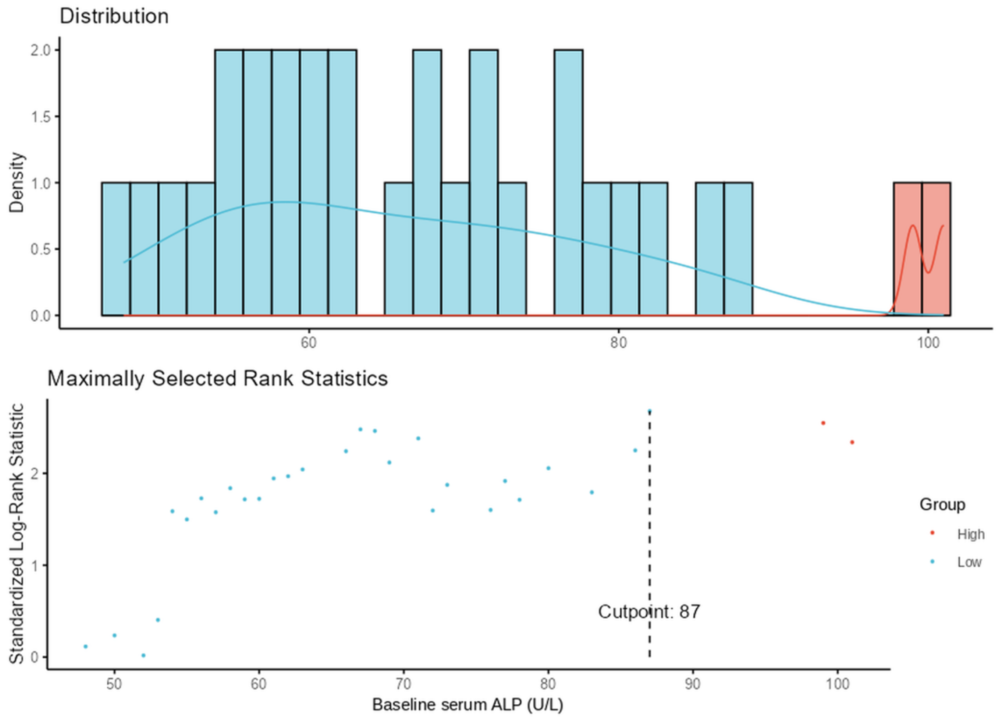

Figure S3. Determination of the optimal cut-off value for baseline serum alkaline phosphatase (ALP) using maximally selected rank statistics. Panels as in Figure S1. The dashed line marks the selected cut-off (87.0 U/L; maximum standardized log-rank statistic = 2.68).

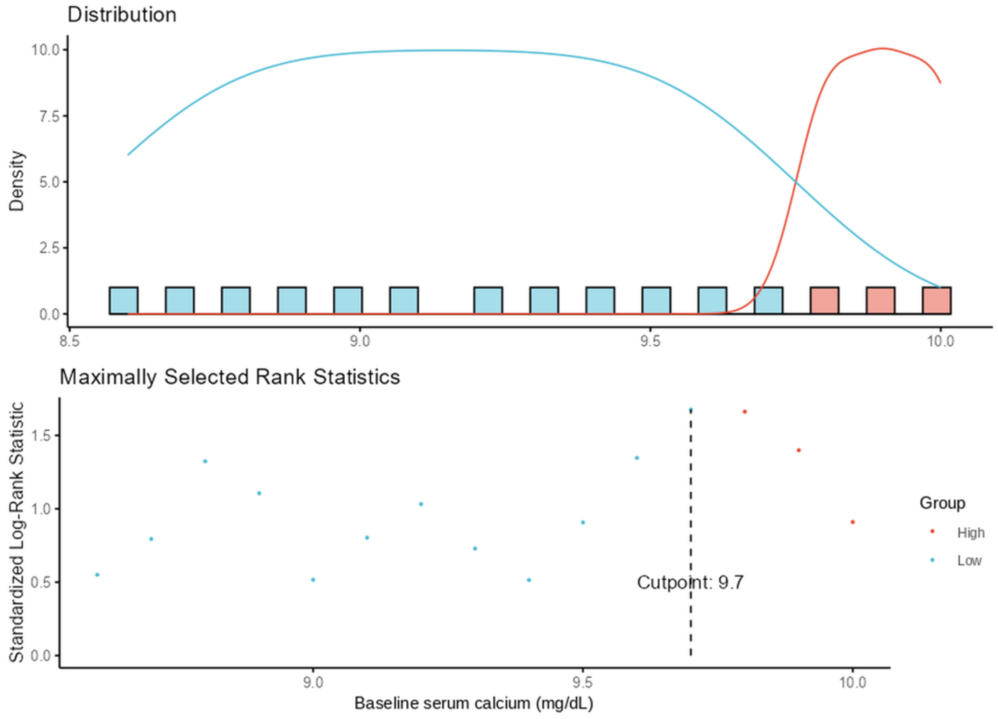

Figure S4. Determination of the optimal cut-off value for baseline serum calcium using maximally selected rank statistics. Panels as in Figure S1. The dashed line marks the selected cut-off (9.70 mg/dL; maximum standardized log-rank statistic = 1.68).

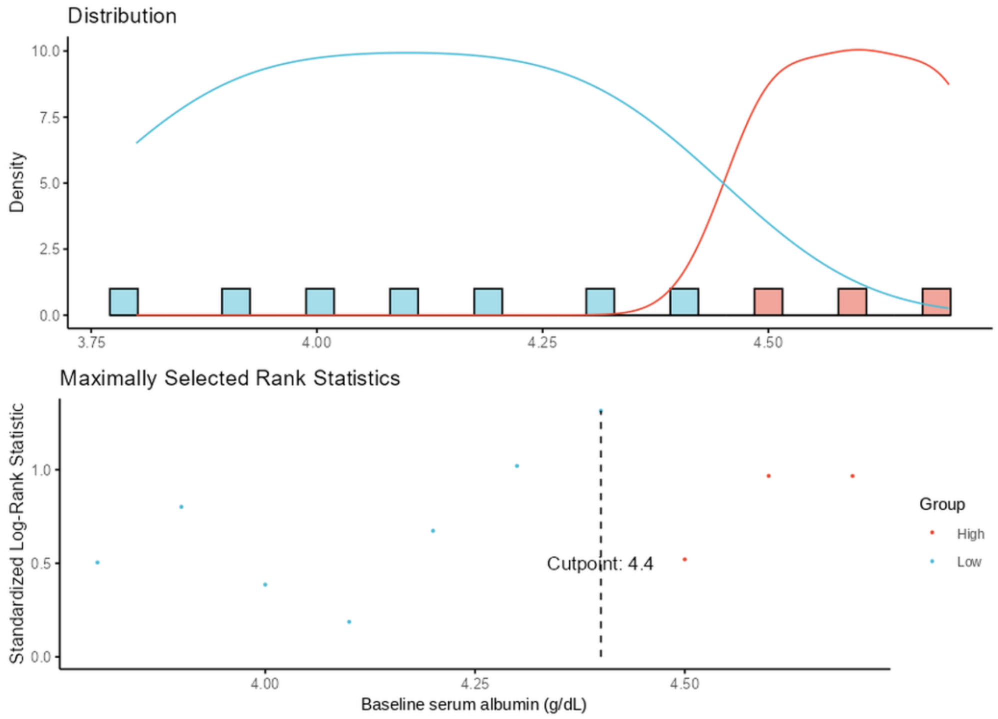

Figure S5. Determination of the optimal cut-off value for baseline serum albumin using maximally selected rank statistics. Panels as in Figure S1. The dashed line marks the selected cut-off (4.40 g/dL; maximum standardized log-rank statistic = 1.31).

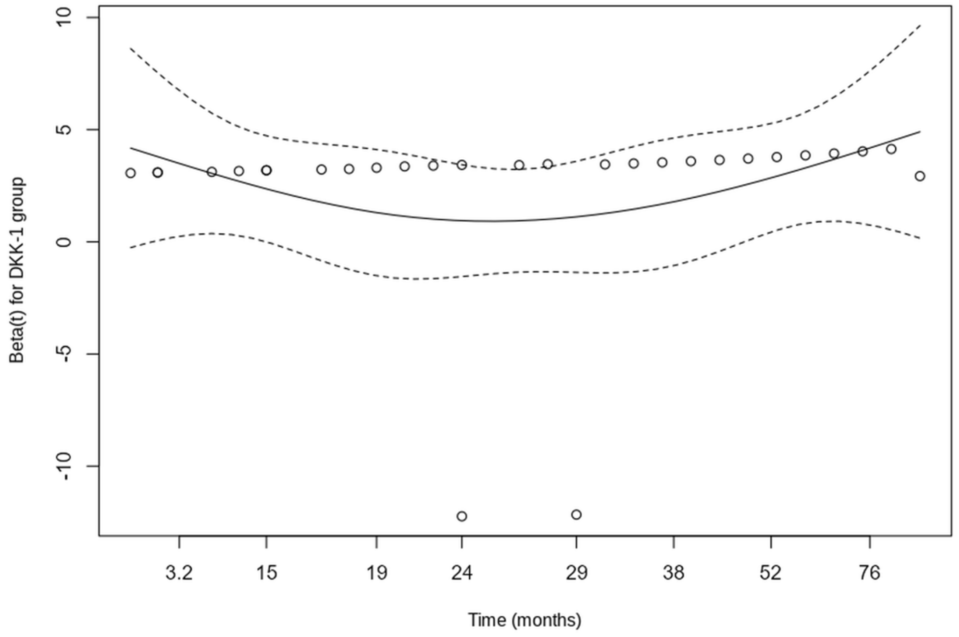

Figure S6. Assessment of the proportional hazards assumption for the DKK-1 group (>66.75 vs. ≤66.75 pg/mL) in the univariable Cox model for bone metastasis-free survival. Scaled Schoenfeld residuals are plotted against time with a smoothed fit (solid line) and its 95% confidence band (dashed lines). The Schoenfeld residual test showed no evidence of violation of the proportional hazards assumption ($\chi^2 = 0.100$, df = 1, p = 0.752).

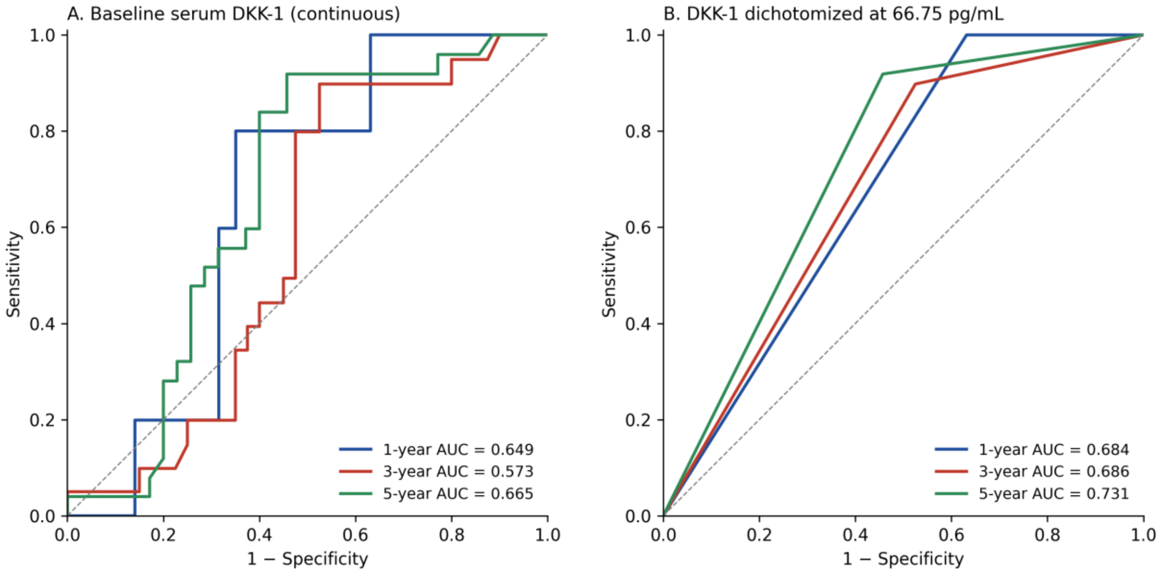

Figure S7. Time-dependent receiver operating characteristic (ROC) curves for bone metastasis at 1, 3, and 5 years for baseline serum DKK-1 as a continuous variable (A) and dichotomized at the 66.75 pg/mL cut-off (B). Cumulative/dynamic ROC curves were estimated with inverse probability of censoring weighting; 95% CIs for the AUCs were obtained by bootstrap resampling (2000 replicates): (A) 1 year, 0.649 (0.454–0.830); 3 years, 0.573 (0.429–0.720); 5 years, 0.665 (0.524–0.804); (B) 1 year, 0.684 (0.624–0.746); 3 years, 0.686 (0.586–0.787); 5 years, 0.731 (0.623–0.830). The 1-year estimates were based on five events, and the AUCs of the dichotomized variable may be optimistic because the cut-off was derived in the same cohort. AUC, area under the curve.
